# Deep Learning Classifies Low- and High-Grade Glioma Patients with High Accuracy, Sensitivity, and Specificity Based on Their Brain White Matter Networks Derived from Diffusion Tensor Imaging

**DOI:** 10.3390/diagnostics12123216

**Published:** 2022-12-19

**Authors:** Sreejith Vidyadharan, Budhiraju Veera Venkata Satya Naga Prabhakar Rao, Yogeeswari Perumal, Kesavadas Chandrasekharan, Venkateswaran Rajagopalan

**Affiliations:** 1 Department of Electrical and Electronics Engineering, Birla Institute of Technology and Science Pilani, Hyderabad Campus, Hyderabad 500078, India; 2 Department of Pharmacy, Birla Institute of Technology and Science Pilani, Hyderabad Campus, Hyderabad 500078, India; 3 Department of Imaging Sciences and Interventional Radiology, Sree Chitra Tirunal Institute for Medical Sciences and Technology, Trivandrum 695011, India

**Keywords:** white matter, connectivity matrix, diffusion tensor imaging, deep neural network, low-grade glioma, high-grade glioma

## Abstract

Classifying low-grade glioma (LGG) patients from high-grade glioma (HGG) is one of the most challenging tasks in planning treatment strategies for brain tumor patients. Previous studies derived several handcrafted features based on the tumor’s texture and volume from magnetic resonance images (MRI) to classify LGG and HGG patients. The accuracy of classification was moderate. We aimed to classify LGG from HGG with high accuracy using the brain white matter (WM) network connectivity matrix constructed using diffusion tensor tractography. We obtained diffusion tensor images (DTI) of 44 LGG and 48 HGG patients using routine clinical imaging. Fiber tractography and brain parcellation were performed for each patient to obtain the fractional anisotropy, axial diffusivity, radial diffusivity, and mean diffusivity weighted connectivity matrices. We used a deep convolutional neural network (DNN) for classification and the gradient class activation map (GRAD-CAM) technique to identify the neural connectivity features focused on by the DNN. DNN could classify both LGG and HGG with 98% accuracy. The sensitivity and specificity values were above 0.98. GRAD-CAM analysis revealed a distinct WM network pattern between LGG and HGG patients in the frontal, temporal, and parietal lobes. Our results demonstrate that glioma affects the WM network in LGG and HGG patients differently.

## 1. Introduction

Glioma, the most common brain tumor type, is generally categorized into low-grade glioma (LGG) and high-grade glioma (HGG). HGG tumors are usually cancerous, and nearly three-fifths of adults are diagnosed with HGG, which has a high mortality and an aggressive growth rate [1,2]. Brain tumors are also widely prevalent in young children [3]. Therefore, an early and accurate diagnosis will help to plan treatment strategies and prognoses.

Usually, tumor grading is performed using biopsy-based histopathological data [4,5]. Biopsy, being an invasive procedure, is prone to surgical complications. Hence, if neuroimaging could aid in accurate tumor diagnosis and grading, the need for biopsy can be minimized. Magnetic resonance imaging (MRI) is predominantly used in brain tumor diagnosis. Among the different MRI modalities, T1-weighted, fluid-attenuated inversion recovery, T2-weighted, and T1-weighted contrast-enhanced images are routinely used to qualitatively identify/classify tumor grades and their shape [6,7,8].

Even though quantitative measures can be extracted from these conventional qualitative images, such as T1-weighted, the correspondence between the derived quantitative measures and underlying pathophysiological changes brought out by the disease process is unclear. Further, previous studies [9,10] used texture and volume features to classify LGG and HGG. Yang et al. [9] used MRI sequences such as T1-weighted, T2-weighted, fluid-attenuated inversion recovery, T1-weighted contrast-enhanced, diffusion weighted imaging, and dynamic contrast-enhanced, etc., for classifying LGG and HGG. They extracted several texture features, namely global, gray-level co-occurrence matrix, gray-level run-length matrix, and gray-level size-zone matrix. Using machine learning, they achieved a training accuracy/AUC score of 0.76/0.86 and a testing accuracy/AUC score of 0.85/0.97. On the other hand, Ding et al. [10] used only T1-weighted contrast-enhanced images and reported an accuracy/sensitivity/specificity of 0.8/0.84/0.76 in classifying LGG and HGG. In the above studies, the accuracy and performance of the machine learning algorithm was moderate.

Therefore, quantitative methods which could capture pathophysiological processes brought out by tumors on the brain’s neural tissue will provide insight. One such MRI sequence developed recently is diffusion tensor imaging (DTI). DTI can provide quantitative measures and allow us to reconstruct virtual neuronal fiber tracts, which can aid in interpreting the underlying neuronal changes brought out by the disease process.

Previous studies [6,11,12,13] investigated the usefulness of DTI measures in classifying LGG from HGG tumors. Quantitative DTI measures, including fractional anisotropy (FA), axial diffusivity (AD), mean diffusivity (MD), and radial diffusivity (RD), were compared in tumor regions such as a solid tumor and peritumoral regions of LGG and HGG patients. Statistically significant differences in DTI measures were observed between the LGG and HGG patients. To mention some of the studies, El-Seoughy et al. [6] found a significant difference between the tumor patients when using the MD measure. In contrast, Jiang et al. [11] reported a significant difference when using AD. Similarly, Piyapittayanan et al. [12] reported a significant difference when using apparent diffusion coefficient values. Inoue T et al. [13] observed lower MD and higher FA values in HGG patients compared to LGG, and Yuan et al. [1] found AD and RD measures to be significantly different between LGG and HGG patients. The reason for the above discrepancy in results between studies is not apparent. We believe that factors such as tumor heterogeneity in LGG and HGG groups, data sample size, the accuracy of tumor segmentation, MR imaging parameters, and differences in image processing steps/software used may have contributed to the differences. Further, the proportion of white matter (WM) present in the tumor and its constituents may also influence the DTI metrics. For instance, in our LGG and HGG patients, we have observed (unpublished) that the glioma tumors predominantly occupy grey matter tissue when compared to WM, so the DTI measures may not correctly reflect the differences between the tumor groups.

The above-mentioned DTI studies have considered brain tumors as local phenomena. However, Sharifi et al. [2] showed that glioma propagates to other brain regions through the corpus callosum. In the review article, Daffau [3] concludes that tumors spread through WM, with myelination affecting tumor migration, suggesting the importance of studying connectomics in glioma patients to understand tumor progression and therapeutic targets. Sharifi et al.’s [2] and Daffau’s [3] results suggest that the WM connectomes outside the tumor region may play a vital role in the glioma disease process and must be investigated. Further, understanding differences in brain connectomics between LGG and HGG patients may aid in treatment planning and strategies. Therefore, this study aims to (i) investigate the differences in the entire brain connectomics between LGG and HGG patients derived from DTI based on their FA, AD, MD, and RD connectivity/adjacency matrices using deep neural networks (DNN) and (ii) use the MRI data *from routine clinical* diffusion weighted imaging protocol (usually of low resolution and anisotropic voxels) which reflects the realistic scenario (i.e., in a clinical setting it is not possible to acquire high-resolution diffusion weighted data in a large number of directions due to various constraints).

## 2. Materials and Methods

### 2.1. Data Acquisition

MRI scans of 92 brain tumor patient data (44 LGG and 48 HGG) were acquired from Sree Chitra Tirunal Institute of Medical Science and Technology, Thiruvananthapuram, India. Inclusion criteria include histopathologically proven cases, where the engineer is blind to histopathological results. Exclusion criteria include cases where all the mentioned sequences were unavailable: DTI, axial T2-weighted, fluid-attenuated inversion recovery, T1-weighted, susceptibility weighted imaging, T1-weighted contrast-enhanced, and fast spin echo T2-weighted images. Recruitment was conducted by the radiologist. The ground truth was the post-surgical histopathological result of the tumor, which can be high grade or low grade. Before surgery, the radiologist (one of the authors) classified the tumor as high or low grade based on radiological features in each case. The study was conducted according to the Declaration of Helsinki. The internal ethics committee at the hospital approved this study (IEC Regn No. ECR/189/Inst/KL/2013/RR-16). The approval number is IEC/1177. The internal ethics committee waived patient informed consent due to the retrospective nature of this study. All procedures were performed under relevant guidelines.

### 2.2. Imaging Protocol

The patients were scanned using a 1.5 T Siemens MRI scanner (Magnetom Avanto, Erlangen, Germany). The imaging parameters include single-shot echo planar imaging (SS-EPI) sequence along 20 or 30 diffusion-weighted (b = 1000 s/mm^2^) directions (since the data are chosen from a routine clinical retrospective database) and one b = 0 s/mm^2^, in-plane resolution = 512 × 448, repetition time (TR) = 3500 ms, echo time (TE) = 105 ms.

### 2.3. Data Processing

#### 2.3.1. Diffusion Tensor Image Processing

**Figure 1:**
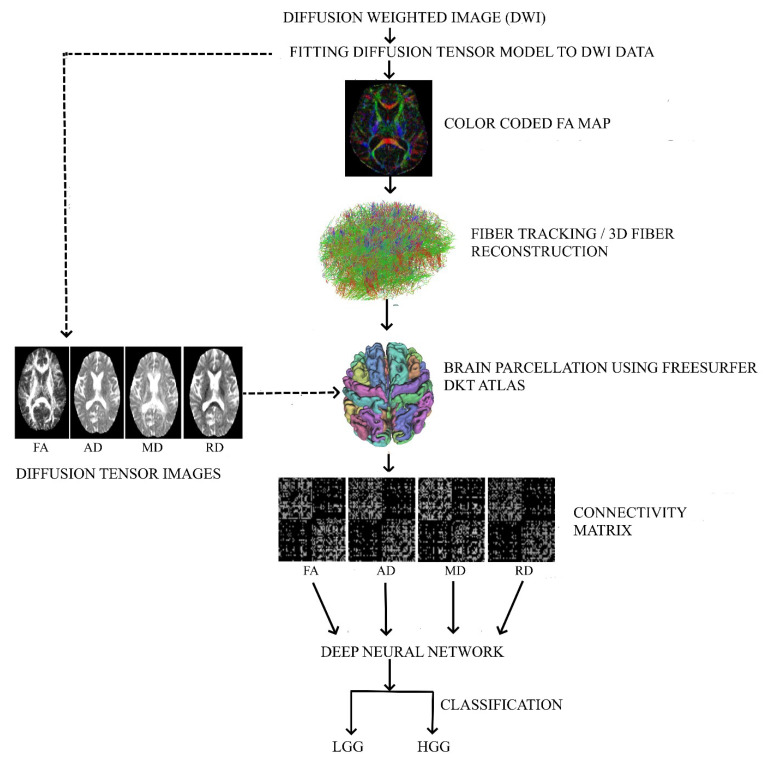
Various steps/stages of data processing are shown in the workflow diagram. These steps/stages include fitting the diffusion tensor model to the diffusion weighted data, fiber tracking, constructing the connectivity matrix for all the DTI (FA, AD, MD, and RD) measures, and implementing the DNN model for classification.

Diffusion weighted images were processed using DSI-Studio (30 December 2020, build https://dsi-studio.labsolver.org/download.html). The image processing steps include motion artifact correction followed by brain segmentation to exclude the non-brain voxels and generation of four connectivity matrices of FA, AD, MD, and RD for each patient with the following steps performed in DSI-Studio: (i) In the first stage, we fitted the diffusion tensor model to the raw diffusion weighted data and obtained FA, AD, MD, and RD maps, (ii) in the next stage, we obtained a color-coded FA map. This FA color map has eigenvectors encoding WM fiber directions, (iii) using the color-coded FA map, we applied the deterministic fiber tracking algorithm [14] to generate fiber tracts and implemented augmented tracking strategies to improve reproducibility [15]. A seed region was placed in the WM volume to construct the whole brain fiber tracts. We discarded the tracks with a length shorter than 30 or longer than 200 mm, (iv) in the fourth stage, we performed brain parcellation using the FreeSurfer DKT atlas, (v) in the fifth stage, using the parcellated atlas and the FA, AD, MD, and RD maps from the first stage, the WM connectivity matrices weighted by FA, AD, MD, and RD were obtained, (vi) in the final step, we provided the four WM connectivity matrices of both patient groups as input to the neural networks. The above four kinds of connectivity matrices (.mat file obtained from DSI-Studio) were converted into grayscale images using MATLAB and given as separate data to the neural network’s input layer. Figure 1 shows the workflow details.

#### 2.3.2. Deep Neural Network Model to Classify LGG and HGG

We designed two types of deep neural networks (DNN), one in which we considered the connectivity matrices as images and the other in which the connectivity matrices were considered *as matrices*. We used 368 connectivity matrix images from 92 patients comprising FA, AD, MD, and RD weighted adjacency matrices.

#### 2.3.3. Deep Neural Network Architecture for Connectivity Matrix as Images

**Figure 2:**
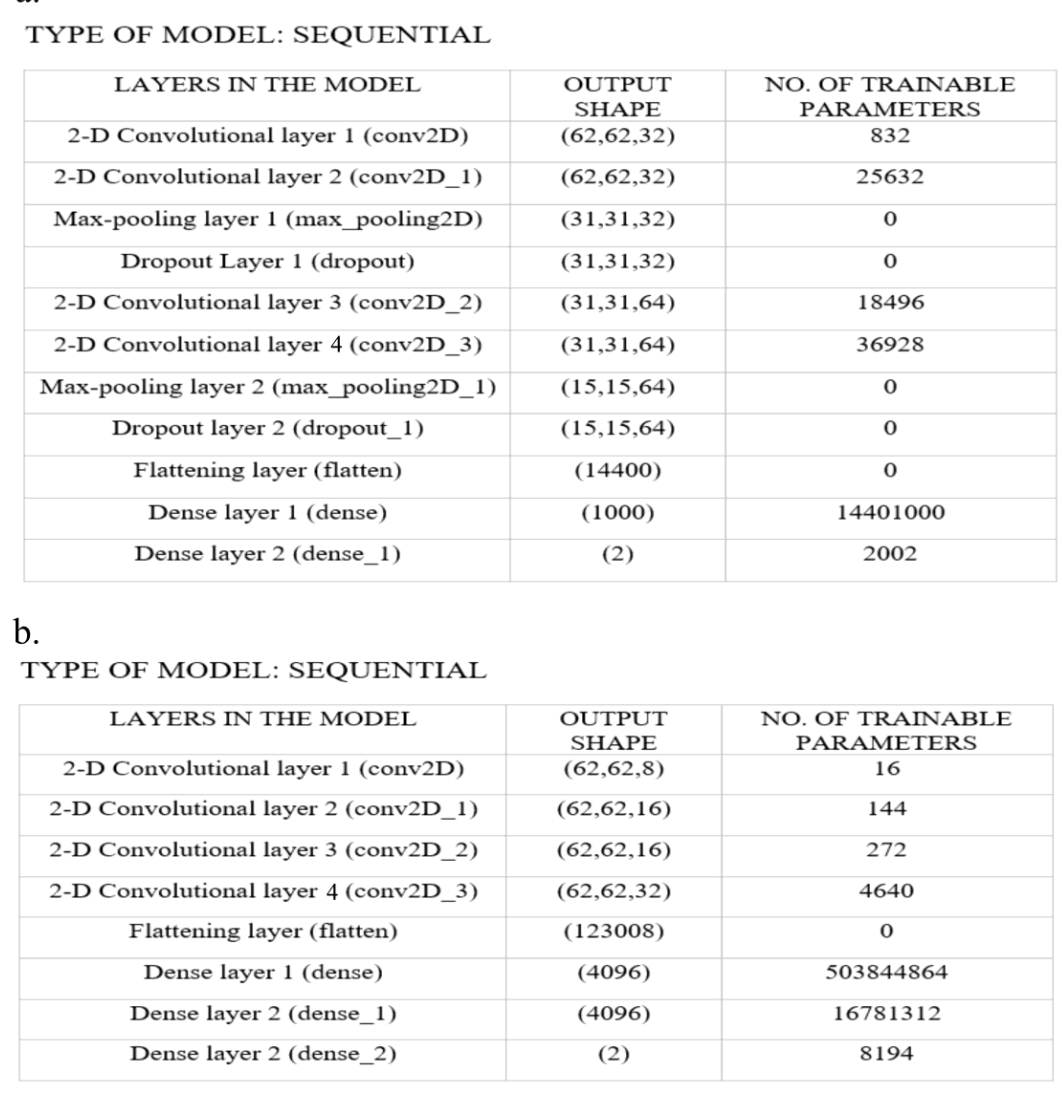
Deep neural network architectures (**a**) 11-layer model designed to classify when connectivity matrix was considered as an image and, (**b**) model with 8 layers where convolutional layers are having kernel size of 1x1 to classify LGG and HGG when their connectivity matrix was considered as a matrix. The model layer details were obtained from TensorFlow.

The connectivity matrix files obtained from DSI-Studio were in mat file format. These matrices were converted into grayscale images using a custom-written MATLAB (2017 version https://in.mathworks.com/products/matlab.html accessed on 24 April 2022) code. We experimented with different DNN architectures by changing the number of convolutional layers (8,4), filter kernel size (16,32,64), number of epochs (10,15,20), and dense layers (1000,2048,4096). We chose the best architecture reported in this study by considering the performance of all these different architectures for classification in terms of accuracy, sensitivity, and specificity. A DNN architecture consisting of 11 layers was implemented to classify LGG and HGG using the connectivity matrix images, as shown in Figure 2a. The first two layers were 2D convolutional layers, each having 32 filters with a kernel size of 3 × 3, followed by a 2D max-pooling layer and a random dropout layer for regularization. This is followed by another set of two 2D convolutional layers, each having 64 filters with a kernel size of 3 × 3, followed by a 2D max-pooling layer and a random dropout layer for regularization. The output from the last convolutional layer is flattened and connected to a fully connected layer. The fully connected layer consists of a hidden layer with 1000 neurons and an output layer of 2 neurons. The other network hyperparameters include the batch size of 15, epochs = 12, Adam optimizer, and RELU activation function was used in all layers, except the output layer, where softmax activation function was used. We considered 70% of the data for training and 30% for testing. Five-fold cross-validation was performed. Custom Python code was written to implement the above architecture using TensorFlow library (Python version 3.9.7, https://www.tensorflow.org/ accessed on 15 June 2022).

#### 2.3.4. Deep Neural Network Architecture Considering the Connectivity Matrix as a Matrix

In the second approach, we considered the connectivity matrix as it is (i.e., as a matrix and not as an image). For this analysis, we adopted the DNN model by Yasaka et al. [16]. This DNN comprises four convolutional layers with 8, 16, 16, and 32 filters whose kernel size was 1 × 1. The purpose of choosing the filter kernel of size 1 × 1 and not having a max-pooling layer is to consider the connectivity matrix as it is and not as an image. The convolutional layers were followed by three fully connected layers consisting of 4096, 4096, and 2 neurons. The architecture detail is given in Figure 2b. Other network hyperparameters include the batch size of 10, epochs = 10, and AdaGrad optimizer for training. The RELU activation function was used for the convolutional layers, and softmax was used for the output classification layer. Custom Python code was written to implement the above architecture using TensorFlow library (Python version 3.9.7, https://www.tensorflow.org/ accessed on 15 June 2022).

The purpose of considering the connectivity matrix as a matrix in this DNN architecture is to visualize and understand the brain network connectivity features used/focused on by the DNN for the classification task. To visualize and extract the regions focused on by the DNN on the connectivity matrix, the gradient class activation map (GRAD-CAM) technique was used [17]. The GRAD-CAM was implemented using TensorFlow and Keras in an open-source Python code (https://pyimagesearch.com/2020/03/09/grad-cam-visualize-class-activation-maps-with-keras-tensorflow-and-deep-learning/ accessed on 10 September 2022). Briefly, the GRAD-CAM processing steps include: (a) The last convolutional layer was used as the target layer. (b) The loss associated with DNN’s prediction for both the classes (LGG, HGG) was used in computing the gradients by the automatic differentiation function of TensorFlow. (c) The weights obtained using the gradient calculation were multiplied with the respective feature maps [17]. A heatmap image was obtained for each class (LGG, HGG) by summing the weighted feature maps from the previous step. We considered the top 18 regions with high values in the GRAD-CAM heatmap for both classes (LGG, HGG).

## 3. Results

### 3.1. Performance of the DNN Model on Connectivity Matrix as Image

**Figure 3:**
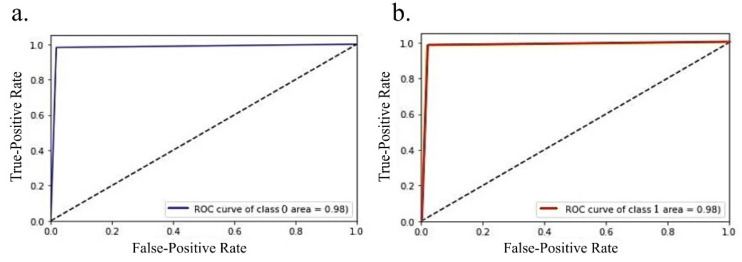
Receiver operating characteristics of the deep learning model where connectivity matrix was considered as an image for LGG (shown in (**a**)) and HGG (shown in (**b**)), the area under curve (AUC) score for both classes were 0.98. The above plot was obtained using Python sklearn package.

We achieved a training accuracy of 0.96 (the model was trained using 258 images) and a testing accuracy of 0.98 (110 images were used for testing). The receiver operating characteristic curve (ROC) is shown in Figure 3; the area under the curve value is 0.98. The confusion matrix for the test data is shown in Table 1. Only one sample in each patient group was incorrectly classified. The evaluation metrics of the convolutional neural network model are shown in Table 2.

### 3.2. Performance of the DNN Model on the Connectivity Matrix as it is

**Figure 4:**
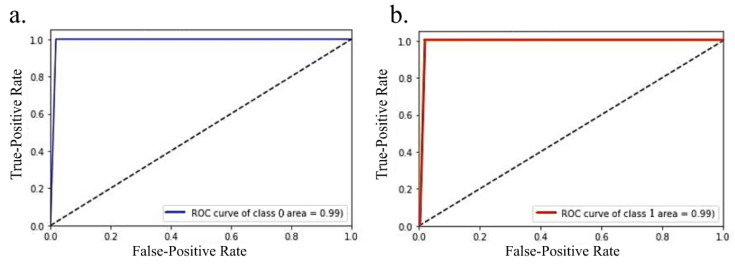
Receiver operating characteristics of the deep learning model where connectivity matrix was considered as a matrix for LGG (shown in (**a**)) and HGG (shown in (**b**)), the area under curve (AUC) score for both classes were 0.99. The above plot was obtained using Python sklearn package.

A training accuracy of 0.97 (the model was trained using 258 images) and a testing accuracy of 0.99 (110 images were used for testing) were achieved. The receiver operating characteristic curve (ROC) is shown in Figure 4; the area under the curve value is 0.99. The confusion matrix for the test data is shown in Table 3. All the LGG patients were correctly classified, and only 1 HGG sample was incorrectly classified. The evaluation metrics of the DNN model are shown in Table 4.

### 3.3. GRAD-CAM Results

**Figure 5:**
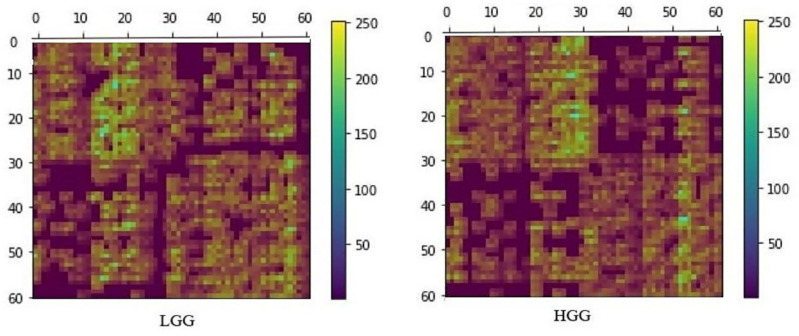
A typical heatmap of an LGG (**left**) and an HGG (**right**) patient is shown. This heatmap was obtained from the trained DNN where the connectivity matrix was considered as a matrix. To illustrate, the heatmap was superimposed on a FA connectivity matrix image from a typical LGG and HGG patient. The 0-61 axis values refer to 62 ROIs from DesikanDKT Atlas (refer to the supplementary table for more details of the ROIs). The color scale depicts the heatmap values.

**Figure 6:**
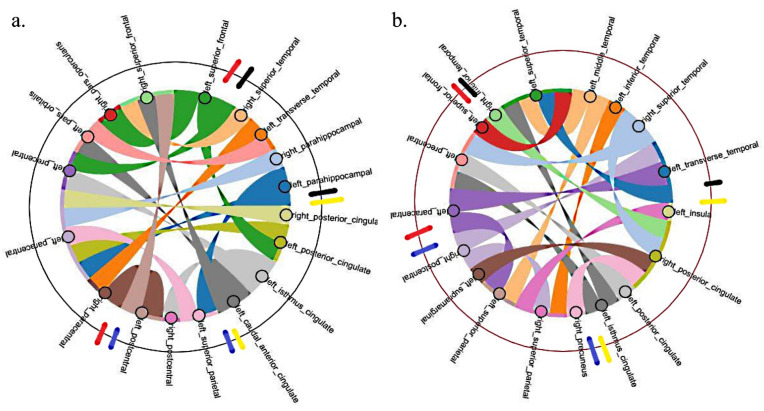
Shows top 18 (in terms of highest GRAD-CAM heatmap values) WM neural connections considered from the respective heatmaps of LGG and HGG patients. (**a**) LGG (**left**) (**b**) HGG (**right**). Legends in red (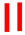) shows frontal lobe regions, black (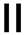) shows temporal regions, yellow (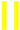) shows cingulate cortex, and blue (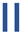) shows parietal regions of the brain. The chord plot was developed using the HoloViews package (Python version 3.9.7). The color of the arcs/ribbons in the chord plot only shows whether the connections are present among different brain ROIs and do not depict the connection strength. left- left brain hemisphere, right- right brain hemisphere.

A typical heatmap that shows the regions in the connectivity matrix focused on by the DNN obtained from the GRAD-CAM analysis for a typical LGG and HGG patient is shown in Figure 5. The top 18 (in terms of highest heatmap values) neural connections considered from this heatmap to understand the WM network features extracted by the DNN for classification are shown in Figure 6.

## 4. Discussion

The main findings of this study include: (a) DNN classifies LGG and HGG with very high accuracy, sensitivity, and specificity based on their WM network connectivity, (b) the WM network connectivity pattern appears to be distinct between LGG and HGG, (c) GRAD-CAM analysis shows significant differences in the brain regions focused on by the DNN for classifying LGG and HGG, i.e., the DNN used/focused on more of the WM network underlying frontal lobe regions in LGG and more of the temporal, parietal WM network regions in HGG.

The diffusion metrics reflect the microscopic pathophysiological changes of a neuron, i.e., changes in FA value indicate WM integrity [18], damage to axons is reflected in AD [19], changes in MD value suggest damage to WM tissue reflecting edema [20], the inflammation process, and RD reflects damage to myelin [19]. So, we considered the adjacency matrix weighted by the above DTI measures for these reasons. Initially, we trained our DNN models separately for each of these adjacency matrices (i.e., DNN model considering only the FA adjacency matrix data, DNN model considering only the AD adjacency matrix, etc.). The accuracy was below 0.7 for these DNN models when independently trained with FA, AD, MD, and RD connectivity matrices. The reasons for this may be (a) changes caused by the glioma disease process in these DTI measures, when considered independently, may not have reached a level that is sufficiently different for the DNN to classify LGG and HGG, and (b) the sample size needed for training and testing was probably not sufficient for the DNN when considering the above DTI measures separately (only 92 connectivity matrices for both training and testing). Hence, we pooled the connectivity matrices from all the above DTI measures. This approach resulted in high accuracy, sensitivity, and specificity while classifying the LGG and HGG patients. Reasons for this may be (a) FA, AD, MD, and RD capture different pathophysiological aspects of neuronal degeneration. It appears that this complementary information is required to classify LGG and HGG glioma patients, and (b) the optimal data sample size requirement for the DNN is probably met.

The DNN results for both conditions, i.e., (a) when the connectivity matrix was considered as an image and (b) connectivity matrix as a matrix, was remarkable (superior accuracy, sensitivity, and specificity values). This demonstrates that DNN could learn and detect significant differences in the neural connections between the LGG and HGG patients. The robustness of our above DNN architectures was verified using a five-fold cross-validation method. In each fold, we consistently observed the same accuracy value of 0.98 (DNN architecture for connectivity as an image) and 0.99 (DNN architecture for connectivity as a matrix).

Whether the WM network connections between LGG and HGG are considered an image or a matrix, the DNN could classify the patient groups with very high accuracy. This demonstrates that the glioma process affects the brain WM network connectivity between LGG and HGG differently. To identify those WM network connections focused on by the DNN to classify LGG and HGG, the information from the DNN feature maps need to be visualized. Therefore, the GRAD-CAM technique was used to extract the features focused on by the DNN. For this purpose, we considered the DNN model we designed by considering the connectivity matrix *as a matrix* where we had a kernel size of 1 × 1 with no max-pooling [16].

Figure 5 shows the heatmap obtained from the GRAD-CAM technique for LGG and HGG, where the axis represents 62 (0–61) parcellated brain regions obtained from the FreeSurfer DKT atlas. The yellow regions (heatmap value range 150–250) are the ones that the DNN highly emphasizes for classification. We considered these highly focused ROIs top 18 neural connections by setting a threshold > 150 from the heatmap. These 18 neural connections for LGG and HGG are shown in Figure 6. The connectome (seen in Figure 6) shows that in the case of LGG patients, out of the top 18 features focused on by the DNN, 7 ROIs are in the frontal lobe, 4 in the temporal lobe, 4 are in the cingulate cortex, and 3 regions from the parietal lobe. Similarly, in HGG patients, the features focused on by the DNN include three ROIs from the frontal lobe, six in the temporal lobe, four in the cingulate cortex, and five regions from the parietal lobe. The above results suggest that in the case of LGG patients, for radiological diagnosis and for planning clinical treatment strategies, the frontal lobe ROIs, namely right paracentral, left paracentral, left precentral, left pars orbitalis, right pars opercularis, right superior frontal, and left superior frontal can be focused on when compared to HGG. Similarly, in the case of HGG patients, the temporal and parietal lobe ROIs, namely right inferior temporal, left superior temporal, left middle temporal, left inferior temporal, right superior temporal, left traverse temporal, right precuneus, right superior parietal, left superior parietal, left supramarginal, and right postcentral can be focused on for radiological and clinical evaluation. The WM neural connectivity pattern difference due to the glioma disease process between the LGG and HGG patients in the above ROIs has enabled the DNN to focus on these ROIs for classification. The occipital lobe regions did not appear in the top 18 features in either LGG or HGG, suggesting that the occipital lobe may not be distinctly affected between the two patient groups.

Previous studies [21,22,23] on WM structural connectivity in brain tumor patients followed different approaches. Liu et al. [21] used a statistical inference (t-test, correlation, etc.) approach to compare brain tumor patients using FA, fiber length, and fiber number weighted connectivity matrices. On the other hand, Yu et al. [22] performed graph-theory-based WM network connectivity analysis in tumor patients. Zhong et al. [23] employed a tract statistics approach using FA, MD, and streamline count weighted connectivity matrices to classify glioblastoma patients. Instead of the statistical inference approaches followed in the above studies, we focused on statistical learning. Hence, a direct comparison cannot be made. However, in terms of brain regions that were found to be affected in LGG and HGG patients, we can find some commonalities between ours and theirs.

Liu et al. [21] observed reduced FA values in the left precentral gyrus (frontal lobe), right supplementary motor area (frontal lobe), left inferior parietal lobule (parietal lobe), right angular gyrus (parietal lobe), right superior orbitofrontal cortex (frontal lobe), right calcarine (occipital lobe), right parahippocampal gyrus (temporal lobe), and right insula (cingulate cortex) in glioma patients. Yu et al. [22] reported seventeen different brain ROIs affected in the parietal, temporal, occipital, frontal lobes, and cingulate cortex. Zhong et al. [23] also observed WM degeneration in the parietal, temporal, occipital, frontal lobes, and cingulate cortex using a tract statistics approach. In line with the above studies, we have also observed WM network connectivity differences in frontal, parietal, temporal, occipital lobes, and cingulate cortex in our glioma patients. The brain regions that were commonly found to be affected between our study and the previous studies in glioma patients include precentral regions in the frontal lobe [21,22,23], parahippocampal in the temporal lobe [21,22,23], superior parietal region in the parietal lobe [22], inferior and superior frontal regions in frontal lobe [22], traverse temporal region in the temporal lobe [23], and the paracentral region in frontal lobes [23].

Previous DTI studies [11,24,25,26] in glioma patients primarily focused on evaluating the DTI measures (FA, AD, MD, and RD) in the tumor, its constituents, and the normal-appearing WM outside the tumor region. On the other hand, we focused on the entire brain’s WM network weighted by these DTI measures using fiber tractography across different brain regions. This approach gives more insight into the WM connectivity pattern in LGG and HGG patients, which the global DTI metric changes in the tumor and its surrounding WM tissues cannot provide. Histopathological studies are needed to confirm the WM connectivity differences detected by the DNN between LGG and HGG. Our results using the routine clinical MRI dataset with low resolution, can be confirmed by future studies that employ high-resolution data with isotropic voxels.

## 5. Conclusions

Glioma affects the WM network of LGG and HGG patients. DNN can classify the LGG and HGG patients with high accuracy, sensitivity, and specificity based on the differences in their WM networks. Therefore, the WM networks obtained using diffusion tensor tractography can help the radiologist to classify LGG and HGG tumors.

## Figures and Tables

**Table 1:** The confusion matrix for the DNN model where the connectivity matrix was used as an image. A total of 110 test images were used.

	PREDICTED		
ACTUAL	GROUP	LGG	HGG
	LGG	54	1
	HGG	1	54

**Table 2:** Evaluation metrics’ precision, recall, specificity, and sensitivity for the DNN model where the connectivity matrix was used as an image is shown (values are given up to four decimal places to track even slight differences in the model’s performance).

Group	TP	TN	FP	FN	Precision	Recall	Specificity	F1-Score
LGG	54	54	1	1	0.9818	0.9818	0.9818	0.9818
HGG	54	54	1	1	0.9818	0.9818	0.9818	0.9818

TP = True Positive, FP = False Positive, TN = True Negative, FN = False Negative. Precision = TP/(TP + FP), Recall/Sensitivity = TP/(TP + FN), Specificity = TN/(TN + FP), F1-score = 2TP/(2TP + FP + FN).

**Table 3:** The confusion matrix for the DNN model where the connectivity matrix was used as a matrix. A total of 110 test images were used.

	PREDICTED		
ACTUAL	GROUP	LGG	HGG
	LGG	55	0
	HGG	1	54

**Table 4:** Evaluation metrics’ precision, recall, specificity, and sensitivity for the DNN model where the connectivity matrix was used as a matrix is shown (values are given up to four decimal places to track even slight differences in the model’s performance).

Group	TP	TN	FP	FN	Precision	Recall	Specificity	F1-score
LGG	55	54	1	0	0.9821	1	0.9818	0.9909
HGG	54	55	0	1	1	0.9818	1	0.9908

TP = True Positive, FP = False Positive, TN = True Negative, FN = False Negative. Precision = TP/(TP + FP), Recall/Sensitivity = TP/(TP + FN), Specificity = TN/(TN + FP), F1-score = 2TP/(2TP + FP + FN).

## Data Availability

The data used in this study are property of Sree Chitra Tirunal Institute of Medical Sciences and Technology and, therefore, cannot be shared.

## Supplementary Materials

The following supporting information can be downloaded at: https://www.mdpi.com/article/10.3390/diagnostics12123216/s1, Table S1: “DesikanDKT Atlas parcellated brain regions with 0-61 nodes representing the x and y axis of the connectivity matrix”.
