# Supplementary material for: Deep Learning Classifies Low- and High-Grade Glioma Patients with High Accuracy, Sensitivity, and Specificity Based on Their Brain White Matter Networks Derived from Diffusion Tensor Imaging
Source: Diagnostics (Basel). 2022 Dec 19;12(12):3216. doi: 10.3390/diagnostics12123216 (PMC9777902; doi:10.3390/diagnostics12123216)
Supplement: Supplementary file 1 [file diagnostics-12-03216-s001.zip › diagnostics-2025989-SI.pdf]

diagnostics

MDPI

SUPPLEMENTARY TABLE

Table S1. DesikanDKT Atlas parcellated brain regions are given from 0-61 nodes representing the x and y axis in the connectivity matrix.

NODES(0-62)	ROI
0	left_caudal_anterior_cingulate
1	left_caudal_middle_frontal
2	left_cuneus
3	left_entorhinal
4	left_fusiform
5	left_inferior_parietal
6	left_inferior_temporal
7	left_isthmus_cingulate
8	left_lateral_occipital
9	left_lateral_orbitofrontal
10	left_lingual
11	left_medial_orbitofrontal
12	left_middle_temporal
13	left_parahippocampal
14	left_paracentral
15	left_pars_opercularis
16	left_pars_orbitalis
17	left_pars_triangularis
18	left_pericalcarine
19	left_postcentral
20	left_posterior_cingulate
21	left_precentral
22	left_precuneus
23	left_rostral_anterior_cingulate
24	left_rostral_middle_frontal
25	left_superior_frontal
26	left_superior_parietal
27	left_superior_temporal
28	left_supramarginal
29	left_transverse_temporal
30	left_insula
31	right_superior_temporal
32	right_caudal_anterior_cingulate
33	right_caudal_middle_frontal
34	right_cuneus
35	right_entorhinal
36	right_fusiform
37	right_inferior_parietal
38	right_inferior_temporal
39	right_isthmus_cingulate
40	right_lateral_occipital
41	right_lateral_orbitofrontal
42	right_lingual
43	right_medial_orbitofrontal
44	right_middle_temporal
45	right_parahippocampal
46	right_paracentral
47	right_pars_opercularis
48	right_pars_orbitalis
49	right_pars_triangularis
50	right_pericalcarine
51	right_postcentral
52	right_posterior_cingulate
53	right_precentral
54	right_precuneus
55	right_rostral_anterior_cingulate
56	right_rostral_middle_frontal
57	right_superior_frontal
58	right_superior_parietal
59	right_supramarginal
60	right_transverse_temporal
61	right_insula

left- brain left hemisphere

right - brain right hemisphere
